# Effects of Prolonged Whey Protein Supplementation and Resistance Training on Biomarkers of Vitamin B12 Status: A 1-Year Randomized Intervention in Healthy Older Adults (the CALM Study)

**DOI:** 10.3390/nu12072015

**Published:** 2020-07-07

**Authors:** Eva Greibe, Søren Reitelseder, Rasmus L. Bechshøft, Jacob Bülow, Grith W. Højfeldt, Simon R. Schacht, Mads L. Knudsen, Inge Tetens, Marie S. Ostenfeld, Ulla R. Mikkelsen, Christian W. Heegaard, Ebba Nexo, Lars Holm

**Affiliations:** 1 Department of Clinical Medicine/Clinical Biochemistry, Aarhus University Hospital, Palle Juul-Jensens Boulevard 99, DK-8200 Aarhus N, Denmark; ebbanexo@rm.dk; 2 Institute of Sports Medicine Copenhagen, Department of Orthopaedic Surgery M, Bispebjerg Hospital, Nielsine Nielsens Vej 11, DK-2400 Copenhagen NV, Denmark; s.reitelseder@gmail.com (S.R.); r.bechshoeft@gmail.com (R.L.B.); jacob.buelow.02@regionh.dk (J.B.); grithwh@gmail.com (G.W.H.); laho@teamdanmark.dk (L.H.); 3 Department of Biomedical Sciences, University of Copenhagen, Blegdamsvej 3, DK-2200 Copenhagen N, Denmark; 4 Department of Nutrition, Exercise, and Sports, University of Copenhagen, Noerre Alle 51, DK-2200 Copenhagen N, Denmark; simonschacht@nexs.ku.dk (S.R.S.); madslind@nexs.ku.dk (M.L.K.); ite@nexs.ku.dk (I.T.); 5 Arla Foods Ingredients Group P/S, Soenderhoej 10-12, DK-8260 Viby J, Denmark; mstos@arlafoods.com (M.S.O.); ulrmk@arlafoods.com (U.R.M.); 6 Department of Molecular Biology and Genetics, Aarhus University, Gustav Wieds Vej 10, DK-8000 Aarhus, Denmark; cwh@mbg.au.dk; 7 School of Sport, Exercise and Rehabilitation Sciences, University of Birmingham, 142 Edgbaston Park Road, Birmingham B15 2TT, UK

**Keywords:** vitamin B12, cobalamin, intervention, whey, whey protein hydrolysate, carbohydrate, maltodextrin, collagen, collagen protein hydrolysate, exercise, fasting versus non-fasting blood samples

## Abstract

We investigated the effect of long-term whey supplementation on biomarkers of B12 status in healthy older adults subjected to different schemes of supplements and exercise. The total study population examined at baseline consisted of 167 healthy older adults (age ≥ 65 year) who were randomized to 1-y intervention with two daily supplements of (1) whey protein (3.1 µg B12/day) (WHEY-ALL), (2) collagen (1.3 µg B12/day) (COLL), or (3) maltodextrin (0.3 µg B12/day) (CARB). WHEY-ALL was comprised of three groups, who performed heavy resistance training (HRTW), light resistance training (LITW), or no training (WHEY). Dietary intake was assessed through 3-d dietary records. For the longitudinal part of the study, we included only the participants (*n* = 110), who met the criteria of ≥ 50% compliance to the nutritional intervention and ≥ 66% and ≥ 75% compliance to the heavy and light training, respectively. Fasting blood samples collected at baseline and 12 months and non-fasting samples collected at 6 and 18 months were examined for methylmalonic acid, B12 and holotranscobalamin. At baseline, the study population (*n* = 167) had an overall adequate dietary B12 intake of median (range) 5.3 (0.7–65) µg/day and median B12 biomarker values within reference intervals. The whey intervention (WHEY-ALL) caused an increase in B12 (*P* < 0.0001) and holotranscobalamin (*P* < 0.0001). In addition, methylmalonic acid decreased in the LITW group (*P* = 0.04). No change in B12 biomarkers was observed during the intervention with collagen or carbohydrate, and the training schedules induced no changes. In conclusion, longer-term daily whey intake increased plasma B12 and holotranscobalamin in older individuals. No effect of intervention with collagen or carbohydrate or different training regimes was observed. Interestingly, the biomarkers of B12 status appeared to be affected by fasting vs. non-fasting conditions during sample collection.

## 1. Introduction

Low vitamin B12/cobalamin (B12) status is estimated to affect up to 15% of people over the age of 60 years [1,2]. B12 deficiency may cause macrocytic anemia and subacute combined degeneration [1,3,4]. At an advanced age, an impaired B12 status may be caused by a lost ability to produce the gastric intrinsic factor needed for the intestinal uptake of B12. An impaired status can also be caused by low B12 intake and/or a reduced ability to release the vitamin from its carrier proteins in the food during digestion [1,5]. 

B12 status can be assessed by measuring total plasma concentrations of B12 and/or plasma B12 bound to its transport protein transcobalamin (holotranscobalamin, holoTC). The latter signifies the fraction of total plasma B12 available for tissue uptake [2,5]. In addition, methylmalonic acid (MMA) is an important diagnostic tool as this metabolite accumulates in the blood during cellular B12 insufficiency [2,5].

Because of the high prevalence of biomarkers suggesting a subclinical B12 deficiency in older adults [1,2], it is important to identify dietary sources of highly available B12. Epidemiological dietary surveys indicate that the B12 status in humans is positively correlated to the intake of dairy products [6,7,8]. In cow′s milk, B12 is evenly distributed between the whey and the casein micelle fraction, bound to transcobalamin in the whey, and via coordination to histidine residues of the caseins, respectively [9,10]. Compared to other foods, whey (and whey protein isolate) is a readily available source of B12 with high bioavailability, which may be attributed to the easily digestible properties of whey proteins [10,11,12]. In 2017, Dhillon et al. showed that eight weeks daily intake of whey protein isolate improved biomarkers of B12 status in Australian older adults with subclinical B12 deficiency [13]. We recently showed that whey or milk provided over four weeks was as efficient as synthetic B12 supplements in improving biomarkers of B12 deficiency in lactovegetarians [14]. However, the long-term effects of whey and alike supplements and interventions on biomarkers of B12 status in healthy older adults remain to be elucidated. 

In the current study, we explored and compared the effect of a 1-year intervention with two daily supplements of 30 g containing different B12 amounts, whey (3.1 µg B12/day), collagen (1.3 µg B12/day), or carbohydrate (0.3 µg B12/day), on the biomarkers of B12 status in a healthy older Danish population. The effect of the whey supplementation was also studied in relation to different resistance training regimes.

## 2. Materials and Methods 

### 2.1. Participants and Study Design

**Figure 1:**
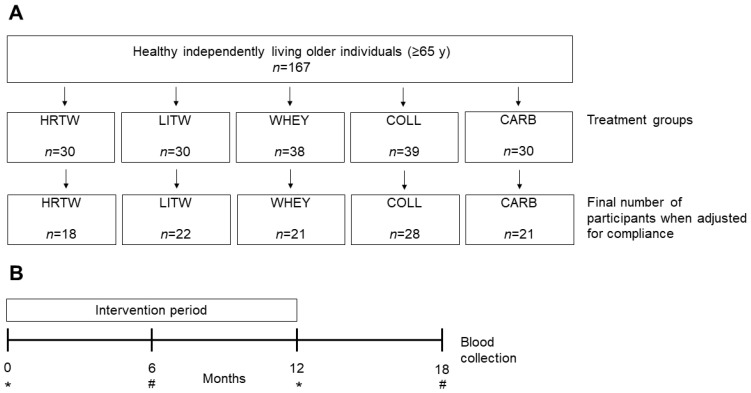
Study design. (**A**) Healthy home-dwelling older individuals were included in the study. The participants were divided in five groups who received two daily nutrient supplements with either whey (WHEY), collagen (COLL), or carbohydrates (CARB) without training intervention or whey supplementation in combination with different training schemes. The heavy resistance training (HRTW) group performed supervised heavy resistance training three times per week in a fitness center. The light resistance training (LITW) group performed supervised home-based light resistance training three-to-five times per week. For the longitudinal part of the study, we included data from participants in the nutrition-only groups (WHEY, COLL, and CARB) with adherence ≥ 50% and with training adherence in the HRTW and LITW groups of ≥ 66% and ≥ 75%, respectively. In the text, the term “WHEY-ALL” is used for the HRTW, LITW, and WHEY groups combined. (**B**) Blood was collected at baseline (fasted (*)) and after six mo (non-fasted (#)) and 12 mo (fasted (*)) of intervention and again after a six mo follow-up period (18-mo) (non-fasted (#)) and measured for B12 biomarkers.

For the present study, we included blood samples available by May 2018 from *n* = 167 participants at baseline and *n* = 110 participants for the longitudinal part of the study. The number of participants included in the longitudinal study was adjusted according to compliance to the dietary intervention and training schedule (see below). The details for the design are outlined in Figure 1.

All samples were derived from the CALM study, which has previously been described in details [15]. In brief, healthy and independent older subjects (*n* = 209, age ≥ 65 years) were recruited from 2014 to 2017 in the Greater Copenhagen area in Denmark to study age-related loss of skeletal muscle mass in a longitudinal randomized controlled trial (for details, see [15]). The participants were randomized into five groups and prospectively assigned to one year of daily intervention with whey (WHEY), collagen (COLL), or carbohydrate (CARB) supplement without training and in a subset of participants receiving whey in combination with either Heavy Resistance Training (HRTW) or Light Resistance Training (LITW). The total group receiving whey (with and without training) is referred to as WHEY-ALL in the following (for details, see Intervention) [15].

Blood samples were collected at baseline (before intervention) and after six months (6 mo; day 178) and 12 months (12 mo; day 359) of intervention and again after a six months follow-up period (18 mo; day 540) at Bispebjerg Hospital, Copenhagen, Denmark. As part of the original CALM design, overnight fasting blood samples were collected at baseline and 12 mo prior to an oral glucose tolerance test [15]. The samples collected at 6 mo and 18 mo were taken in the afternoon and were non-fasting. Blood (~500 µL) was drawn into tripotassium ethylenediaminetetraacetic acid (K_3_-EDTA) tubes and centrifuged for 10 min at 3220× *g* at 4 °C. Plasma was collected and stored at −80 °C until shipment to the Department of Clinical Biochemistry, Aarhus University Hospital, Denmark, on dry ice for analysis of B12 related biomarkers (see Biochemical Measurements).

The CALM Intervention Study was approved by the Danish Regional Ethics Committee of the Capital Region (project no. H-4-2013-070) and registered at the Danish Data Protection Agency (project. no. 2012-58-0004) and at clinicaltrials.gov (ID NCT02034760). The study was performed within the confines of the Helsinki Declaration II, and all participants gave their informed consent before inclusion.

### 2.2. Intervention

A complete overview of the intervention and study design can be found elsewhere (15). In brief, the participants were given a sachet containing 30 g of supplement twice per day for one year. The sachets contained 10 g sucrose and 20 g of either whey protein hydrolysate (3.1 µg B12/day) (WHEY-ALL), collagen hydrolysate (1.3 µg B12/day) (COLL), or maltodextrin (0.3 µg B12/day) (CARB). All sachets were developed, prepared, and individually packed by Arla Food Ingredients Group P/S, Viby J, Denmark. The content of B12 in the sachets were analyzed by Eurofins using a Biacore instrument. In brief, B12 was extracted with a phosphate buffer at pH 4.5 with added cyanide to convert all B12 forms to dicyano-B12. The total amount of dicyano-B12 was determined by an inhibition assay on the Biacore using the Qflex Vitamin B12 kit (Biacore, Uppsala, Sweden) in which the competing protein was a modified form of the B12-binding protein haptocorrin.

In total, three groups received whey supplements. One of the groups performed supervised heavy resistance training three times per week in a fitness center (HRTW). Another group performed supervised home-based light resistance training 3–5 times per week (LITW). The last group received no training intervention (WHEY). The detailed exercise regimes for the HRTW and LITW groups can be viewed in [15].

The intake of supplements and the adherence to light intensity training were self-reported using hardcopy diaries. In the HRTW group, every training session was supervised, and the staff recorded adherence. We included data from participants in the nutrition-only groups (WHEY, COLL, and CARB) with adherence ≥50% (corresponding to ≥20 g protein/maltodextrin + ≥10 g sucrose) and with training adherence in the HRTW and LITW groups of ≥ 66% (corresponding to ≥2 times per week) and ≥75% (corresponding to ≥3 times per week), respectively.

### 2.3. Biochemical Measurements

Plasma was analyzed for the B12 biomarkers MMA, B12, and holoTC concentrations at the Department of Clinical Biochemistry at Aarhus University Hospital, Denmark. All samples from each participant were analyzed in one run. MMA was quantified by Liquid Chromatography–Tandem Mass Spectrometry on the AB SCIEX Triple Quad 5500 System (AB SCIEX). Plasma B12 was measured on the Advia Centaur CP Immunoassay System (Siemens) after a 1:2 dilution in 0.9% NaCl. HoloTC was determined by an in-house sandwich ELISA after removal of unsaturated transcobalamin with B12-coated magnetic beads [16]. Out of the cohort, some of the plasma samples contained a limited volume not sufficient for measurement of all three biomarkers. In these cases, we prioritized the analysis of the biomarkers in the following order: MMA, B12, and holoTC. The number of samples measured are indicated in figures, tables, and text, whenever relevant.

Hemoglobin, mean red blood cell volume (MCV), and creatinine were measured at the Department of Clinical Biochemistry at Bispebjerg Hospital, Denmark. Hemoglobin and MCV were determined on the Sysmex XN 9000 (Sysmex). Creatinine was determined on the Cobas 8000 (Roche).

### 2.4. Intake of B12 from Diets and Supplements

After careful instructions from staff, study participants undertook a complete weighed dietary record for three consecutive days (Wednesday to Friday) at baseline and again after 11 mo of the intervention. All dietary information was entered by trained staff into the electronic dietary assessment tool, VITAKOST (2018-version, VITAKOST Aps, Kolding, Denmark), for estimation of study participants′ individual dietary B12 intake using the Danish Food Composition Databank (Version 7.01, The National Food Institute, DTU, Kgs. Lyngby, Denmark) to assess nutrient intake. B12 intake from supplements was estimated from the content of the supplements and the median adherence rate to the supplement. A full description of nutrient intakes and adequacies in the CALM study population has previously been reported [17].

### 2.5. Statistical Analysis

The D’Agostino–Pearson omnibus test was used to test whether data followed the Gaussian distribution. Fasting blood samples (baseline vs. 12 mo) and non-fasting blood samples (6 mo vs. 18 mo) were analyzed separately. For each type of blood samples, an overall multivariate analysis of variance model was used for each biomarker to allow for multiple comparisons. Statistical differences between fasting blood samples and between non-fasting blood samples for the three biomarkers were estimated subsequently with the paired t-test or the Wilcoxon signed-rank test (non-normal data). Comparisons of absolute concentrations and delta-values between groups were estimated with the one-way ANOVA with Tukey′s post hoc corrections or with the Kruskal–Wallis test with Dunn′s post hoc corrections (non-normal data). Values of *P* < 0.05 were accepted as statistically significant. The data analysis was performed by using the statistical software available in GraphPad Prism version 7.03 (GraphPad, La Jolla, CA, USA).

## 3. Results

### 3.1. Characteristics of the Study Population

We included 167 healthy home-dwelling Danish older citizens (89 males and 78 females) aged (median (range)) 69 (65–82) years and dispersed in the following age categories: 65–69 years (*n* = 84), 70–74 years (*n* = 60), 75–79 years (*n* = 19), and > 80 y (*n* = 4). The population weighed (median (range)) 73 (50–122) kg and had a height of 172 (154–192) cm. Median (range) baseline concentrations of creatinine (80 (48–108) µmol/L), hemoglobin (9.0 (7.3–11.1 mmol/L), and MCV (89 (81–100 fL) were within reference intervals [18,19]. Biomarkers of B12 status at baseline are presented in Table 1. Median values of MMA, B12, and holoTC are within reference intervals of adults [16,20,21]. No sex-specific differences in biomarker levels were found.

The longitudinal study covered the participants (*n* = 110) who met the compliance criteria for the nutritional and training interventions (see Figure 1). The baseline parameters in the individual groups were comparable to the findings for the whole cohort (presented in Table 1), and there were no differences between the groups at baseline for any biomarker or in age, weight, or height.

### 3.2. Dietary B12 Intake

At baseline, the participants (*n* = 167) had a daily dietary B12 intake of (median (range)) 5.3 (0.7–65) µg. The participants in the longitudinal study (*n* = 110) had a comparable daily intake of B12 at baseline of 5.2 (0.9–65) µg, and there was no difference in the reported B12 intake between any of the intervention groups at baseline *P* = 0.21) or at 11 mo (*P* = 0.11) as judged by the Kruskal–Wallis test.

The intervention supplements (two sachets of 30 g per day) supplied an additional amount of B12 (whey: 3.1 µg B12/day; collagen: 1.3 µg B12/day; and carbohydrate: 0.3 µg B12/day). The participants in the longitudinal study (*n* = 110) had a median compliance to the nutritional intervention of 93%, which provides an estimate of a total daily median B12 intake of ~ 8 µg (WHEY-ALL), ~ 6 µg (COLL), and ~ 5 µg (CARB) during the 12-mo intervention period. These intake levels are similar to the mean intake range observed for several European countries [22] and well above the Estimated Average Requirement of 2.0 µg/day set by Institute of Medicine, US [23].

### 3.3. Effect of Whey Intervention on Biomarkers of B12 Status

**Figure 2:**
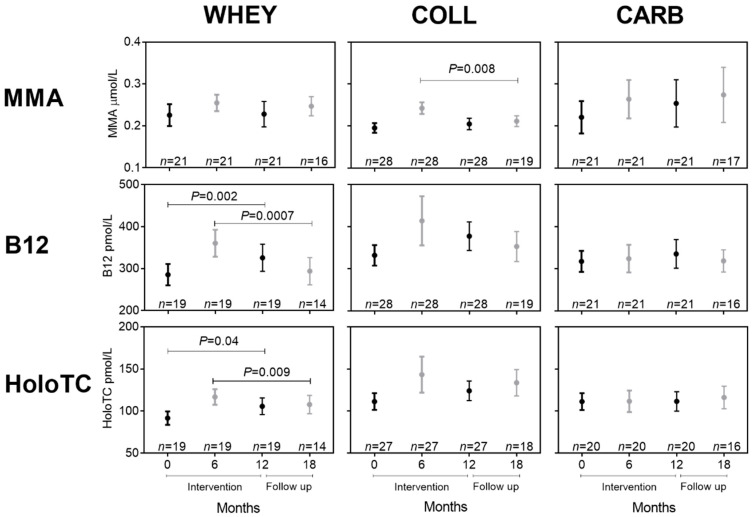
Changes in markers of B12 status in response to nutritional intervention. Healthy older participants received 1 year of intervention with whey (WHEY), collagen (COLL), or carbohydrate (CARB) with no additional training. Plasma concentrations of MMA, B12, and holoTC at baseline and after six months (6 mo) and 12 months (12 mo) of intervention and again after a six months follow-up period (18 mo) are shown as means with their standard errors. The number of observations is indicated (*n* = x). Plasma samples were obtained in the overnight fasted state at baseline and 12 mo (black symbols), where the nutritional state was not controlled at 6 mo and 18 mo (grey symbols). Therefore, the statistical comparisons are made pairwise within each of the states, and the statistical differences between fasting blood samples (baseline vs. 12 mo, black) and between non-fasting blood samples (6 mo vs. 18 mo, grey) were estimated with the paired t-test or the Wilcoxon signed-rank test (non-normal data). Abbreviations: B12, vitamin B12; holoTC, holotranscobalamin; MMA, methylmalonic acid.

First, we looked at the effect of the nutritional supplementation in non-training groups. Figure 2 shows the markers of B12 status before, during, and after the intervention and again after a six months follow-up period for the three nutritional intervention groups that received supplements with whey (WHEY), collagen (COLL), and carbohydrate (CARB).

Initial comparisons of the results from fasting samples (baseline and 12 mo) with non-fasting samples (6 mo and 18 mo) showed differences that may be explained by the differences in fasting status. When comparing the mean values of all fasting samples (all participants at baseline and 12 mo) with the mean values of all non-fasting samples (all participants at 6 mo and 18 mo), we observed that the fasting samples were statistically lower than the non-fasting samples for MMA (*P* < 0.0001) and holoTC (*P* = 0.049). The same numerical tendency was observed for B12 (*P* = 0.16). Therefore, in the subsequent comparisons, we limited our analysis to compare the results obtained for fasting samples (baseline vs. 12 mo) and the results obtained for non-fasting samples (6 mo vs. 18 mo).

We found an increase in B12 and holoTC in response to 1-y whey intervention (WHEY) but no change in MMA (Figure 2, 0 vs. 12 mo). Six months after the supplementation had been discontinued, B12 and holoTC had declined (6 mo vs. 18 mo). Data for the whey groups combined (WHEY-ALL, *n* = 61) showed a comparable picture (Supplementary Figure S1). No change in plasma B12, holoTC, or MMA occurred in the COLL and CARB groups in response to one year of intervention (Figure 2). However, comparing results at 6 mo and 18 mo showed a decline in MMA for the COLL group (*P* = 0.008). Nevertheless, overall, there was no statistical difference between the nutritional groups for any of the biomarkers after 12 months of intervention or after the six months follow-up period (18 mo) as judged by the Kruskal–Wallis test on both absolute concentrations and on delta-values.

### 3.4. Effect of Training in Combination with Whey Intervention on Biomarkers of B12 Status

**Figure 3:**
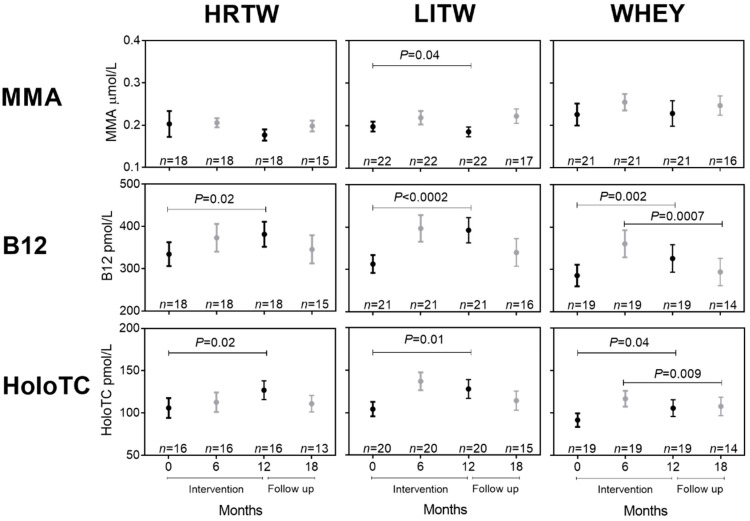
Changes in markers of B12 status in response to training intervention in combination with whey supplementation. Healthy older participants received one year of intervention with whey in combination with heavy resistance training (HRTW), light resistance training (LITW), or no added training (WHEY). Plasma concentrations of MMA, B12, and holoTC at baseline and after six months (6 mo) and 12 months (12 mo) of intervention and again after a six months follow-up period (18 mo) are shown as means with their standard errors. The number of observations is indicated (*n* = x). Plasma samples were obtained in the overnight fasted state at baseline and 12 mo (black symbols), where the nutritional state was not controlled at 6 mo and 18 mo (grey symbols). Therefore, the statistical comparisons are made pairwise within each of the states, and the statistical differences between fasting blood samples (baseline vs. 12 mo, black) and between non-fasting blood samples (6 mo vs. 18 mo, grey) were estimated with the paired t-test or the Wilcoxon signed-rank test (non-normal data). Abbreviations: B12, vitamin B12; holoTC, holotranscobalamin; MMA, methylmalonic acid.

Next, we looked at the effect of different training regimes in combination with whey supplementation on the markers of B12 status, shown in Figure 3, by comparing the HRTW, LITW and WHEY groups. The participants in the HRTW and LITW groups had a median compliance to the training regimes of 82%.

B12 and holoTC increase in response to one year of whey supplementation for all three whey groups (HRTW, LITW, and WHEY) (Figure 3, baseline vs. 12 mo). In absolute terms, the numerically largest changes after one year of intervention (baseline vs. 12 mo) were seen for the LITW group with a mean ∆B12 of 80 pmol (26% increase) and a mean ∆HoloTC of 24 pmol/L (23% increase). In contrast, the HRTW group showed a mean ∆B12 of 47 pmol (14% increase) and a mean ∆HoloTC of 21 pmol/L (20% increase), and the WHEY group showed a mean ∆B12 of 40 pmol (14% increase) and a mean ∆HoloTC of 14 pmol/L (15% increase). However, these changes were not statistically different between the groups as judged by the Kruskal–Wallis test on the delta-values or on the absolute concentrations after 12 months of intervention or after the six months follow-up period. Nevertheless, as support for the trend seen for B12 and holoTC, a decrease in MMA concentrations (mean ∆MMA of 0.0122 µmol/L; 6% decrease) (Figure 3) was seen only in the LITW group ((*P* = 0.04).

## 4. Discussion

Here, we investigated and compared the effect of one year supplementation with two daily doses of whey (3.1 µg B12/day), collagen (1.3 µg B12/day), and carbohydrate (0.3 µg B12/day) and different training regimes on the biomarkers of B12 status in a cohort of healthy Danish older adults (age ≥ 65 y). We report three findings: (1) Fasting conditions influence the concentration of B12 biomarkers; (2) baseline biomarkers of B12 status were relatively low despite a daily B12 intake far above recommendation; and (3) biomarkers of B12 status were improved by whey supplementation, irrespective of addition of training.

The study has some limitations. Blood at baseline and 12 mo was collected in the morning as fasting samples, whereas blood at 6 mo and 18 mo was collected in the afternoon as non-fasting samples. These different conditions were mirrored in the biomarker results and limited our analysis to pairwise comparisons of results of time points, baseline vs. 12 mo (fasting) or 6 mo vs. 18 mo (non-fasting). However, this limitation in the design paved the road for an important finding. We observed that the fasting samples in general gave rise to lower concentrations of MMA and holoTC than the non-fasting samples. The same statistically insignificant trend was observed for B12. Our findings are surprising as B12 and holoTC previously have been found to be insensitive to fasting state and food intake [24,25], and most laboratories do not require fasting before assessment of B12 status. Our study points to the importance of considering fasting state when investigating B12 metabolism. One limitation of the study is that data on intake of nutritional supplements was not collected. However, despite this, the study still provides important dietary insights on the effects of daily whey intake in an elderly population.

At the time of inclusion, 16% of this cohort of self-reliant Danish older individuals, had fasting baseline B12 blood concentrations below 200 pmol/L (the lower limit of the reference interval) [21], 3% had holoTC values below 40 pmol/L (the lower limit of the reference interval) [16], and 10% had MMA values above 0.28 µmol/L (the upper limit of the reference interval) [20]. Out of the 16% (*n* =1 4) with low B12, only five showed increased levels of MMA. In addition, we also recorded increased levels of MMA in six participants with normal levels of B12. MMA may be spuriously increased in individuals with an impaired kidney function; however, all of the participants with an increased level of MMA showed normal levels of creatinine. MMA is considered the most specific biomarker for judging B12 status, superior to measures of B12 [1]. Thus, we judge that the participants with increased MMA may well have an impaired B12 status.

The overall pattern of 16% showing plasma B12 concentrations below reference intervals correlates with the finding in other studies on the general Western elderly population [1,2]. Still, our results are surprising since we show that the daily dietary B12 intake (median ~ 5 µg) was twice as high as the RDA of 2.4 µg/day B12 [23] for adults and close to the estimated daily intake of ~6 µg shown to normalize all of the B12 biomarkers (21). Our study does not allow us to conclude whether the finding at baseline relates to an age-related malabsorption of food-bound B12.

The median dietary B12 intake is also higher than the Adequate Intake (AI) value of 4 µg/day set by European Food Safety Authority (EFSA) for adults [22]. Interestingly, we observed an increase in B12 and holoTC after supplementation with whey containing an additional dose of 3.1 µg B12/day for one year. The results support that a significant subset of the elderly have a reduced capacity for absorbing food-derived B12, a capability previously questioned in several studies [2,26,27]. It remains to be documented whether the high proportion of older adults showing biomarkers indicating a suboptimal B12 status simply indicates the need for age stratified intervals of references and/or a stratification in the recommended dietary sources of the vitamin according to the intestinal bioavailability of B12.

The positive effect of whey supplementation on biomarkers of B12 status supports the findings in a recent paper by Dhillon et al. (2017) [13], where older adults with biomarkers indicating subclinical B12 deficiency received whey protein isolate for eight weeks containing ~ 3 µg B12/day and thus underscores the benefit of improving B12 status by B12-containing foods, such as whey.

We found no change in biomarkers after supplementation with collagen (baseline vs. 12-mo). This result could be driven by two factors. First, the daily amount of B12 supplied from the collagen sachets (1.3 µg B12/day) was smaller than from the whey sachets (3.1 µg B12/day), and second, B12 present in milk is considered to have a superior bioavailability compared with B12 present in collagen [11,12]. For future studies, it could be interesting to administer equal doses of B12 in whey, collagen, and carbohydrate (and even other relevant foods) to elucidate the role of the food matrix on the bioavailability of B12 from different food sources.

We did not find an overall effect of training in combination with whey supplementation on the biomarkers of B12 status. The light training (LITW) group showed a higher increase in plasma B12 and holoTC than the heavy training (HRTW) and no training (WHEY) groups as judged from the absolute plasma concentrations. However, this difference was not statistically significant. The only statistically significant difference observed was for MMA, the metabolite of intracellular B12 status in plasma, which decreased significantly more in the LITW group than in the other whey-supplemented groups. The effect of exercise on the biomarkers of B12 status has only been explored in a few studies. In a study by Herrmann et al. (2005) [28] in a younger cohort, recreational athletes (median age 38 y) were found to have an altered B12 metabolism compared with inactive controls [28]. The athletes were found to have higher concentrations of MMA and holoTC than the inactive controls but comparable concentrations of B12. In contrast, Nynke de Jong et al. [29] did not find an effect of exercise on MMA concentrations in frail elders (≥ 70 years). More studies are needed to explore the relationship between exercise and B12 metabolism.

## 5. Conclusions

In conclusion, long-term daily whey intake increased plasma concentrations of B12 and holoTC in older healthy individuals. No increases were observed for intervention with collagen or carbohydrate, and no differences were observed between different training regimes in combination with whey supplementation. Notably, random use of fasting and non-fasting blood samples is to be avoided in longitudinal studies of B12 biomarkers.

## Figures and Tables

**Table 1:** Plasma markers of B12 status at baseline ^1^.

Marker	Reference Intervals	Participants *n* = 167
MMA µmol/L	0.08–0.28	0.19 (0.11–1.60) *n* = 167
B12 pmol/L	200–600	301 (98–740) *n* = 161
HoloTC pmol/L	40–150	93 (22–264) *n* = 156

^1^ Baseline MMA, B12, and holoTC were measured on plasma samples from 167 healthy elderly. Results are presented as medians with (range). Reference intervals are from [16,20,21]. Because of limited volume in some samples, not all biomarkers could be measured in all samples and were prioritized in the following order: MMA, B12, and holoTC. The number of analyzed samples is indicated (*n* = x). At baseline, 16% had fasting baseline B12 blood concentrations below the lower limit of the reference interval. For holoTC, this figure was 3%. For MMA, 10% had values above the upper limit of the reference interval at baseline. This pattern correlates with the finding in other studies on the general Western elderly population [1,2]. Abbreviations: B12, vitamin B12; holoTC, holotranscobalamin; MMA, methylmalonic acid.

## Supplementary Materials

The following are available online at https://www.mdpi.com/2072-6643/12/7/2015/s1, Figure S1. Biomarkers of B12 status in the WHEY-ALL group.
