# Supplementary material for: Effects of Prolonged Whey Protein Supplementation and Resistance Training on Biomarkers of Vitamin B12 Status: A 1-Year Randomized Intervention in Healthy Older Adults (the CALM Study)
Source: Nutrients. 2020 Jul 7;12(7):2015. doi: 10.3390/nu12072015 (PMC7400943; doi:10.3390/nu12072015)

Effects of prolonged whey protein supplementation and resistance training on biomarkers of vitamin B12 status: A 1-year randomized intervention in healthy older adults (the CALM study)

Eva Greibe – Online Supplementary Material

Supplementary Figure 1: Biomarkers of the B12 status in the WHEY-ALL group

Changes in markers of B12 status in response to intervention with whey protein hydrolysate. Healthy older participants received one year of intervention with whey (WHEY-ALL, *n*=61, consisting of *n*=18 performing heavy resistance training, *n*=22 performing light resistance training, and *n*=21 performing no training). Plasma concentrations of MMA, B12, and holoTC at baseline and after six months (6 mo) and 12 months (12 mo) of intervention and again after a six months follow-up period (18 mo) are shown as means with their standard errors. The number of observations are indicated (*n*=x). The statistical differences between fasting blood samples (baseline vs. 12 mo, black symbols) and between non-fasting blood samples (6 mo vs. 18 mo, grey symbols) were estimated with the paired t-test or the Wilcoxon signed-rank test (non-normal data). Abbreviations: B12, vitamin B12; holoTC, holotranscobalamin; MMA, methylmalonic acid.

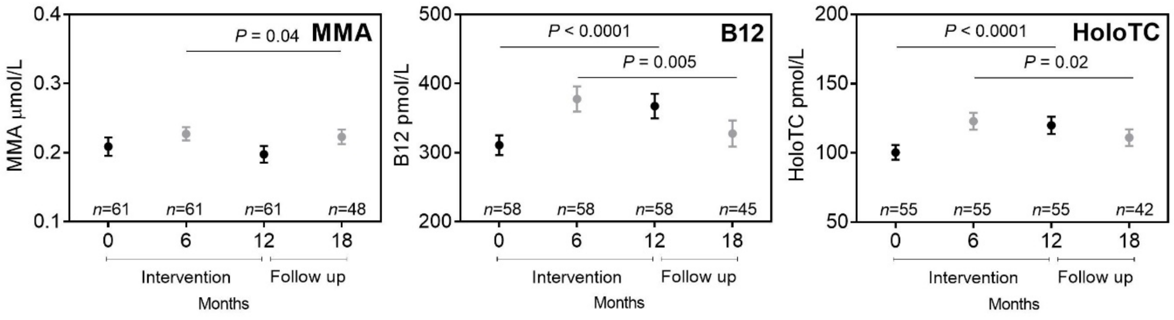

Supplement: Supplementary file 1 [file nutrients-12-02015-s001.pdf]
